# Is there an association between low serum 25-OH-D levels and the length of hospital stay in orthopaedic patients after arthroplasty?

**DOI:** 10.1007/s10195-016-0414-y

**Published:** 2016-06-13

**Authors:** Gerrit Steffen Maier, Uwe Maus, Djordje Lazovic, Konstantin Horas, Klaus Edgar Roth, Andreas Alois Kurth

**Affiliations:** 1 University Hospital of Orthopaedic Surgery, Pius-Hospital, Carl von Ossietzky University, Medizinischer Campus Universität, Georgstrasse 12, 26121 Oldenburg, Germany; 2 Department of Orthopaedic Surgery, Johannes Gutenberg University, Mainz, Germany; 3 ANZAC Research Institute, University of Sydney, Sydney, Australia; 4 Department of Orthopaedic Surgery, Themistocles Gluck Hospital, Ratingen, Germany

**Keywords:** Hypovitaminosis D, Vitamin D deficiency, Length of stay, Orthopaedic patients

## Abstract

**Background:**

The purpose of this observational study was to evaluate serum levels of 25-OH-D in patients scheduled to undergo elective hip or knee arthroplasty. We hypothesised that 25-OH-D level is an independent risk factor for length of stay in orthopaedic patients after elective hip or knee arthoplasty.

**Materials and methods:**

25-OH-D levels were measured in 1083 patients admitted to an orthopaedic surgery department to undergo elective hip or knee arthroplasty. Comparisons were performed using Chi square or Student’s *t* test, followed by univariate and multiple linear regression analysis examining the correlation between the length of stay in the orthopaedic department and 25-OH-D level while adjusting for possible confounders.

**Results:**

Overall, 86 % of patients had insufficient serum levels of 25-OH-D, and over 60 % were vitamin D deficient. The mean length of stay was 13.2 ± 8.3 days. In patients with hypovitaminosis D, the length of stay was significantly longer compared to patients with normal serum 25-OH-D levels (15.6 ± 7.2 compared to 11.3 ± 7.9 days, *P* = 0.014). In univariate analyses, serum 25-OH-D level was inversely related to the length of stay in our orthopaedic department compared to patients with normal vitamin D levels (*r* = −0.16; *P* = 0.008). In multivariate analyses, the length of stay remained significantly associated with low 25-OH-D levels (*P* = 0.002), indicating that low vitamin D levels increase the length of stay.

**Conclusions:**

We found a high frequency of hypovitaminosis D among orthopaedic patients scheduled to undergo elective arthroplastic surgery. Low vitamin D levels showed a significant inverse association to the length of stay in our orthopaedic department. Patients with vitamin D levels in the target range were hospitalised 4.3 days less than patients with hypovitaminosis D.

Level 3 of evidence according to “The Oxford 2011 levels of evidence”.

## Introduction

Vitamin D deficiency is the most common nutritional deficiency worldwide [1]. Variations in vitamin D deficiency prevalence are dependent on the study population, vitamin D fortification of foods, geographic location (mainly latitude) and season [2, 3]. In previous studies, we were able to show a widespread rate of vitamin D insufficiency among elderly orthopaedic patients (aged over 70 years) admitted to an orthopaedic university hospital [4]. Extremely low vitamin D levels have been associated with osteomalacia and impaired muscle function, both core elements in the field of orthopaedic surgery. Good muscle function and healthy bones are essential for fast rehabilitation and positive outcome after orthopaedic surgery, as well as good physical function [4, 5].

Matthews et al. [6] reported an inverse relation between length of hospital stay and vitamin D levels in surgical patients admitted to the intensive care unit. With over 250 patients evaluated, the mean length of stay for patients with severe vitamin D deficiency (<13 ng/mL) was 13.33 days, compared to 5.17 days for patients with vitamin D levels above 27 ng/mL. The length of stay of 253 patients of a geriatric acute care unit was inversely associated with low vitamin D levels. Helard et al. [7] reported that patients with vitamin D levels below 50 nmol/L were hospitalised, on average, 3 days more than patients with vitamin D levels above 50 nmol/L.

We hypothesised that vitamin D level is an independent risk factor for length of hospital stay in orthopaedic patients receiving elective hip or knee arthroplasty.

## Materials and methods

Between 1 January 2011 and 31 December 2012, the serum 25-OH-D levels (referred to as vitamin D) of 1083 patients consecutively admitted to the orthopaedic department of the university hospital in Mainz, Germany (50°N) were measured on admission. Patients were scheduled to receive either elective knee or elective hip arthroplasty. Patients scheduled to receive arthoplastic surgery due to a fracture were excluded form the study. In general, blood was taken on the day of admission. The mean age of the patients was 76 years (± 7.9 years) (Table 1).

**Table 1:** Characteristics and comparison of the participants

	Patients with 25-OH-D <20 ng/mL	Patients with 25-OH-D >20 ng/mL	Significance (*P*)
Sex			
Male (*n* = 516)	391	125	Reference
Female (*n* = 567)	290	277	0.138
Age (years), mean ± SD	76.9	75.5	0.51
Main reason for admission			
Hip arthroplasty (*n* = 606)	370	236	
Knee arthroplasty (*n* = 477)	292	185	
Comorbidities			
Osteoporosis (*n* = 238)	197	41	0.057
Hypertension (*n* = 606)	399	207	0.61
Cardiovascular disease (*n* = 530)	356	174	0.63
Thyreotic Abnormality (*n* = 465)	299	166	0.87
Pulmonary disease (*n* = 205)	130	75	0.52
Renal failure (*n* = 238)	127	111	0.88
Infectious diseases (*n* = 22)	14	8	0.72
Vitamin D Suppl. (*n* = 162)	103	59	0.062
Diabetes (*n* = 509)	244	265	0.89
Obesity (BMI > 30 kg/m^2^) (*n* = 389)	256	133	0.26
Protone pump inhibitors (*n* = 629)	409	220	0.1
Acetylsalicylic acid (*n* = 493)	294	199	0.756
Metamizole (*n* = 328)	200	128	0.19
Diclofenac (*n* = 354)	232	122	0.79
Iso-butyl-propanoic-phenolic acid (*n* = 39)	23	16	0.41
*N*-acetyl-p-aminophenol (*n* = 341)	201	140	0.52
Indomethacin (*n* = 298)	155	143	0.86
Length of stay (days), mean ± SD	15.6 ± 7.2	11.3 ± 7.9	0.014

Measurement of serum 25-OH-D was standardised; the hospital laboratory used the ARCHITECT^®^ 25-OH Vitamin D assay (Abbott GmbH & Co KG, Wiesbaden-Delkenheim, Germany).

Hypovitaminosis D was defined as serum 25-OH-D levels below 20 ng/mL according to the definition of the World Health Organisation [8]. As yet there is no universally accepted classification of vitamin D levels, we defined sufficient vitamin D status as a serum 25-OH-D level of above 30 ng/mL. Vitamin D inadequacy was defined as a serum 25-OH-D level of under 30 ng/mL, and was further divided into vitamin D insufficiency (20–30 ng/mL) and vitamin D deficiency (under 20 ng/mL) as described previously [9].

Patient demographic variables and background data were evaluated by retrospective chart review, and were used as potential confounders. Included variables were age, sex, primary musculoskeletal diagnosis, BMI, comorbidities, oral medication (proton pump inhibitors and analgetics) and any vitamin D supplements taken before admission.

The primary outcome measured in our study was orthopaedic department length of stay. The length of stay was calculated using the administrative registry of the University Hospital of Mainz, Germany, by subtracting day of admission from day of discharge.

Patients’ characteristics were summarised using either means and standard deviations or frequencies and percentages. Firstly, patients were separated into two groups based on vitamin D status (25-OH-D level above 20 ng/mL or below 20 ng/mL).

Comparisons were performed using Chi square or Student’s *t* test, followed by univariate and multiple linear regression analyses examining the correlation between the length of stay in the orthopaedic department (dependent variable) and 25-OH-D level (independent variable) while adjusting for possible confounders. Pearson correlation was used to examine the association between the length of stay in the orthopaedic department and 25-OH-D levels. A *P* value <0.05 was considered to be significant. Statistical analyses was performed with IBM SPSS statistics software (ver. 21; IBM SPSS, Chicago, IL).

## Results

A total of 1083 patients was enrolled in this study; 567 (52.4 %) of the study participants were women, 516 (47.5 %) were men. Mean age was 76 years, with a range from 70 years to 97 years.

Overall, 86 % of the study population was vitamin D insufficient and 63 % of patients were vitamin D deficient. Of the 1083 vitamin D serum levels measured in this study, only 8 % were in the target range of 30 to 60 ng/mL. Serum vitamin D levels of all 1083 patients were normally distributed, with a mean of 17.1 ng/mL; minimum and maximum values ranged from 8 to 78.5 ng/mL, respectively. The mean length of stay was 13.2 ± 8.3 days. We found that the length of stay was longer in patients with hypovitaminosis D compared to patients with normal serum 25-OH-D levels (15.6 ± 7.2 compared to 11.3 ± 7.9 days, *P* = 0.014), the mean difference in length of stay was 4.3 days between groups. There were no other clinical or demographical statistically significant differences. In univariate analyses serum 25-OH-D level was inversely correlated with the length of stay in our orthopaedic department compared to patients with normal vitamin D levels (*r* = −0.16; *P* = 0.008). In multivariate analysis, after adjusting for age, sex, primary musculoskeletal diagnosis, BMI and comorbidities, the length of stay remained significantly associated with low 25-OH-D levels (*P* = 0.002), indicating that low vitamin D levels increase the length of stay (Table 2).

**Table 2:** Multivariate linear regression analyses for association between length of stay and vitamin D deficiency after adjusting for possible confounders

Variables	Mean difference (95 % CI)	Significance (*P*)
Gender (male)	0.01 (−0.1 to 0.3)	0.37
Age	0.09 (−1.5 to 3.28)	0.52
Primary diagnosis (hip arthroplasty vs. knee arthroplasty)	0.48 (−2.83 to 4.01)	0.78
Osteoporosis	0.06 (−4.07 to 3.94)	0.97
Hypertension	0.008 (−0-03 to 0.09)	0.81
Cardiovascular disease	0.5 (−1.4 to 2.4)	0.63
Thyreotic abnormality	0.99 (−2.6 to 2.59)	0.47
Pulmonary disease	0.39 (−5.79 to 5.0)	0.87
Renal failure	0.07 (−4.08 to 3.95)	0.74
Infectious diseases	0.49 (−2.25 to 3.25)	0.72
Diabetes	3.76 (−2.1 to 9.68)	0.2
Obesity (BMI >30 kg/m^2^)	1.26 (−1.42 to 3.95)	0.34
Vitamin D deficiency	4.3 (0.5 to 6.78)	0.002

## Discussion

To the best of our knowledge, this study is the first study to report a possible association between low vitamin D levels and length of stay in orthopaedic patients. Vitamin D status was evaluated in view of its possible role as a predictor of hospital length of stay. We observed a high prevalence of vitamin D-deficient states among patients admitted to the orthopaedic department. Furthermore, a highly significant inverse correlation between 25-OH-D level and length of stay was observed.

Lee et al. [10] demonstrated in a longitudinal cohort study of 191 patients that preoperative hypovitaminosis D was not associated with worse health-related quality of life at 3 months postoperatively. But preoperative hypovitaminosis D had a subtle effect on pain intensity scores in early postoperative period. Furthermore, low vitamin D levels were a risk factor for moderate-to-severe persistent pain after knee arthroplasty. Unnnanuntana et al. [11] evaluated the association between preoperative serum vitamin D level and the attainment of in-hospital functional milestones. Data from 200 patients, scheduled to undergo total hip arthroplasty, was collected. They found no influence of vitamin D on the attainment of in-hospital milestones and the length of stay. Low vitamin D levels were not associated with further perioperative complications. One recent study evaluated the association between serum levels of 25-OD-D and handgrip strength, midupper arm muscle mass and the length of hospital stay after hip fracture; 102 patients with hip fracture and aged over 65 years of age were enrolled. In the multiple linear regression analyses, serum 25-OH-D levels were associated with handgrip strength but not with muscle mass or length of hospital stay [12].

Vitamin D is a steroid hormone with pleiotropic effects [13]. Most of the known biological actions are mediated through binding to the vitamin D receptor present in almost all tissues and cells within the human body. While its classical action is that of a regulator of calcium/phosphate homeostasis and bone metabolism, several studies have demonstrated that this steroid has further multiple roles. Thus, vitamin D is not only important to bone health, but also plays an important role in immunomodulation, regulation of inflammation and cytokines, cell proliferation, cell differentiation, apoptosis, angiogenesis, muscle strength and muscle contraction [6, 9, 14]. Since 25-OH-D is essential for the regulation of cellular growth, differentiation and function, low 25-OH-D levels lead to multiple organ dysfunction, disability and unstable health, which are all causes of deconditioning, polypharmacy and longer length of hospital stay [7, 15, 16]. Several studies revealed a widespread rate of vitamin D insufficiency and deficiency in orthopaedic patients [17–20]. In their study of 723 orthopaedic patients, Bogunovic et al. [17] demonstrated a prevalence of up to 66 % of abnormal (insufficient or deficient) 25-OH-D levels. Patients of dark skin tone were identified to be at higher risk of hypovitaminosis D than those with lighter skin tones. In previous studies, we were able to show a prevalence of 84 % of vitamin D insufficiency, and of 60 % of vitamin D insufficiency in 1119 patients of all ages consecutively admitted to an orthopaedic university hospital in central Germany. Of note, only 15 % of tested patients were in the target range of 30–60 ng/mL [18, 19].

In addition, hypovitaminosis D has been proposed as a new biomarker of longer length of stay in acute care units. Zittermann et al. [21] showed that lower 25-OH-D levels were independently associated with a prolonged intensive care unit stay after cardiac surgery. In further studies, low vitamin D levels doubled the risk of being in hospital for more than 14 days in a geriatric acute care unit [22], and the combination of insufficient 25-OH-D level, male gender and delirium predicted a 4.8-fold higher risk of longer length of stay among geriatric inpatients [23]. Helard et al. [7] evaluated 253 geriatric inpatients of an acute geriatric care unit and showed an inverse linear correlation between serum 25-OH-D level and the length of stay in the acute geriatric care unit. Participants with 25-OH-D levels below 50 nmol/L (20 ng/mL) stayed on average 3 days longer in hospital than those with 25-OH-D above 50 nmol/L. A 1-day decrease in length of stay was seen per 7.1 nmol/L increase in 25-OH-D. In their study of 259 consecutive admitted patients to a surgical intensive care unit, Mathews et al. [6] reported an inverse relationship between vitamin D level and the length of stay in the surgical intensive care unit. Furthermore, vitamin D deficiency was correlated inversely with surgical intensive care unit treatment cost and mortality. Consistent with these results, we were able to show an inverse relationship between 25-OH-D level and the length of stay in our orthopaedic department. Patients with 25-OH-D below 20 ng/mL stayed on average 4.3 days longer on the ward than patients with vitamin D levels above 20 ng/mL.

Vitamin D supplementation is safe and simple. The Endocrine Society’s practice guidelines recommend treatment strategies for patients with low serum vitamin D levels depending on age and underlying medical conditions. They recommend that vitamin D deficient adults should be treated with 50,000 IU vitamin D2 or vitamin D3 once a week for 8 weeks, or with ~6000 IU/day vitamin D2 or vitamin D3, followed by maintenance therapy of 1500–2000 IU/day. In obese patients, patients with malabsorption syndromes, and patients on medications affecting vitamin D metabolism, two to three times higher doses (at least 6000–10,000 IU/day vitamin D) are recommended to treat vitamin D deficiency, followed by maintenance therapy of at least 3000–6000 IU/day [24]. Dawson-Hughes et al. [25] recommended an estimated average essential vitamin D supplementation of 800–1000 IU/day (20–25 µg/day) in order to reach a serum 25(OH)D level above 75 nmol/L in the elderly. Significantly higher dosages are required to ensure that virtually all elderly people achieve this level of vitamin D. In their randomised, double-blind, placebo-controlled single centre trial, Amrein et al. [26] showed that even high-dose oral vitamin D corrected low vitamin D concentration without adverse effects. Vitamin D was given orally or via nasogastric tube once at a dose of 540,000 IU followed by a monthly maintenance dose of 90,000 IU. They showed that, among critically ill patients with vitamin D deficiency, supplementation of vitamin D did not reduce hospital length of stay or hospital mortality. But lower hospital mortality was found in the severe vitamin D deficiency subgroup, but, according to the authors, this finding should be considered hypothesis generating.

Nevertheless, potential limitations to our study should be considered. The majority of the tested patients in this study had white skin tones. Given the predisposition of darker skin-toned individuals toward lower 25-OH-D levels, hypovitaminosis D among darker skin-toned orthopaedic patients may be underrepresented in this study [4]. The generalisability of our findings may be limited, even though we treated a mixed population of adult orthopaedic patients with no restriction on age, sex, or admission diagnosis. Furthermore, the geographical localisation of Mainz (50° northern latitude) limits our results to regions around this latitude, e.g. Paris (48°51′N), Vancouver (49°15′N), Calgary (51°3′N) or Kiev (50°27′) or above 50° latitude. Finally, it should be borne in mind that, in some cases, the length of stay depends not only on bioclinical aspects but is influenced by other factors, e.g. human resource employees, insurance guidelines, number of beds available, etc.

In the present study, we found a high frequency of hypovitaminosis D among orthopaedic patients scheduled to undergo elective hip or knee arthroplasty. Low vitamin D levels showed a significant inverse association to the length of stay in our orthopaedic department. Patients with vitamin D levels in the target range were hospitalised 4.3 days less than patients with hypovitaminosis D.

Vitamin D supplementation is safe and simple and may be a possible way to reduce the length of hospital stay after hip and knee arthroplasty, but further randomised controlled trials on the pre- and post-operative impact of vitamin D supplementation may be needed to confirm this hypothesis.
